# Prognostic significance of TRIM59 for cancer patient survival: A systematic review and meta-analysis

**DOI:** 10.1097/MD.0000000000018024

**Published:** 2019-11-27

**Authors:** Min Wang, Ce Chao, Guanghua Luo, Bin Wang, Xianghong Zhan, Dongmei Di, Yongxiang Qian, Xiaoying Zhang

**Affiliations:** a Department of Cardiothoracic Surgery; b Comprehensive Laboratory, The Third Affiliated Hospital of Soochow University, Changzhou, China.

**Keywords:** cancer, carcinoma, meta-analysis, prognosis, TRIM59, tumor

## Abstract

**Background:**

The family of tripartite motif (TRIM) proteins, which includes 80 known TRIM protein genes in humans, play a key role in cellular processes.^[1]^ TRIM59, a member of the TRIM family of proteins, has been reported to be involved in the carcinogenesis of multiple types of tumors. However, the prognostic value of TRIM59 in the survival of tumor patients remains controversial. We therefore conducted a meta-analysis to assess the prognostic significance of TRIM59 in cancer patients.

**Materials and methods:**

PubMed, Embase, VIP, CNKI and Wanfang Data were searched for eligible reports published before September 30, 2018. The hazard ratio (HR) and 95% confidence intervals (CIs) were adopted to estimate the association between TRIM59 and overall survival (OS).

**Results:**

Six studies with 1584 patients were included to assess the effect. The results showed that high levels of TRIM59 were significantly associated with poor OS in cancer patients (HR = 1.43, 95%CI: 1.24–1.66, *P* < .001), indicating that higher TRIM59 expression could be an independent prognostic factor for poor survival in cancer patients.

**Conclusion:**

Our meta-analysis suggests that higher TRIM59 expression predicts poor prognosis in cancer patients, and it may therefore serve as a promising prognostic factor.

## 1 Introduction

TRIM (Tripartite motif) family proteins harbor an RBCC (RING B-box coiled coil) motif, and contain a common N-terminal RING (Really Interesting New Gene) finger domain, one or two B-box motifs, and a coiled-coil region. Humans possess 70 TRIM protein-coding genes.^[1,2]^ Some of these proteins can be defined as E3 ubiquitin ligases as they contain a RING-finger domain, although not all proteins containing a RING-finger domain function as E3 ubiquitin ligases.^[3,4]^ Tripartite motif (TRIM) family proteins, most of which have E3 ubiquitin ligase activities, have various functions in cellular processes including intracellular signaling, development, apoptosis, protein quality control, innate immunity, autophagy, and carcinogenesis.^[5]^ TRIM family members are through recognition, combined with specific target proteins, play the role of RING finger domain dependent ubiquitin ligase, the important regulatory proteins and ubiquitin binding for ubiquitin modification, induce the target proteins into proteasome dependent ubiquitin degradation process, so as to further regulate the target protein involved in many biological processes.^[1]^

TRIM59 (Tripartite motif 59), is a cell-surface molecule. It is located on human chromosome 3, and is a member of the C-XI subfamily of TRIM family proteins. In addition to an RBCC motif, there is also a TM (transmembrane) domain in the C-terminal region of TRIM59, involved in the regulation of intracellular localization of TRIM59.^[1]^ TRIM59 is located on endoplasmic reticulum in cells and is mainly composed of 403 amino acid residues. It was first cloned by Chang in 2002.^[6]^ It is speculated by work energy analysis that TRIM59 may promote the stability of ubiquitin regulatory proteins by mediating the interaction between proteins. TRIM59 was initially described as a potential oncoprotein in 2011,^[7]^ and since then an increasing number of studies have supported the role of TRIM59 as an oncoprotein. TRIM59 has also been reported to be involved in certain types of human cancer, The expression level of TRIM59 has been shown to be upregulated in many cancers including lung cancer, gastric cancer, breast cancer, hepatocellular carcinoma, colorectal cancer, and others.

Based on bioinformatics analysis, Hao found that the expression level of TRIM59 in NSCLC (non-small cell lung cancer) was significantly increased. Further study showed that the patients with high expression of TRIM59 had high mortality and high recurrence rate, shown that the overexpression of TRIM59 suggested poor prognosis. Cox regression analysis showed that TRIM59 was an independent prognostic factor in tumor tissues. Zhan suggested that TRIM59 may promote the proliferation of NSCLC by up-regulating cell cycle associated proteins. Zhang shown that the expression of trim59 in breast cancer cells is up-regulated. In addition, they also found that TRIM59 can activate TGF-β (transforming growth factor β) signaling pathway to promote the proliferation, migration and invasion of breast cancer cells by decreasing the protein expression level of p-smad2. Zhou found that TRIM59 was highly expressed in gastric cancer and was significantly related to the prognosis of the patients base on bioinformatics retrieval and analysis of multiple databases. Furthermore, the expression levels of TRIM59 mRNA and protein in clinical samples, tissue chips and gastric cancer cell lines were detected. It was found that the expression level of trim59 in gastric cancer tissues was higher than that in paracancerous tissues, and the expression level of TRIM59 in gastric cancer cell lines was also significantly higher than that in normal gastric epithelial cells. At the same time, Luo screened out the single nucleotides polymorphism of three TRIM59 in Chinese Han population. The risk of gastric cancer in the subjects with variant (GA-AA) with rs1141023 was higher than that in the subjects with common genotypes. Further analysis showed that the polymorphism of rs1141023 was significantly correlated with tumor stage and lymph node metastasis. Xue detected the high expression of TRIM59 protein in liver cancer tissues of patients with liver cancer, the difference was statistically significant. Further studies showed that the positive expression of TRIM59 protein was closely related to pathological grade, tumor differentiation, vascular invasion and clinical TNM stage, suggesting that trim59 may play an important role in the differentiation, invasion and metastasis of HCC cells. Sun detected TRIM59 in 90 cases of colorectal cancer by qRT-PCR. It was found that the expression of TRIM59 in colorectal cancer tissue was significantly higher than that in non-cancer tissue, and the high expression of TRIM59 was significantly correlated with TNM stage, lymph node metastasis, depth of invasion and distant metastasis. Kaplan-Meier curve showed that the overall survival time of patients with high expression of TRIM59 was significantly lower than that of patients with low expression of TRIM59. In addition, TRIM59 was also studied for high expression in osteosarcoma, prostate cancer, bladder cancer, cervical cancer, and renal cell carcinoma.

The relationship between TRIM59 and carcinogenesis has been described in several in vitro studies, and some studies have clarified the relationship between TRIM59 and the prognosis of one tumor, but not systematically. The aim of our meta-analysis was therefore to provide a quantitative assessment of the prognostic value of TRIM59 in various types of cancer.

## 2 Materials and methods

### 2.1 Search strategy

PubMed, Embase, VIP, CNKI (China National Knowledge Infrastructure) and Wanfang Data were searched for relevant studies that evaluated the prognostic value of TRIM59 in cancer patients. We evaluated studies published up until September 30, 2018. Our search strategy used the text “TRIM59” or “Tripartite motif 59”. Additionally, the referenced articles in relevant literature were also manually reviewed for suitable studies. This study is a meta-analysis, so there is unnecessary to provide an Ethical Approval.

### 2.2 Inclusion and exclusion criteria

Studies were independently screened by 2 authors (Chao and Wang) according to the title, abstract, and type of article. There were no language restrictions added to the literature search. Articles were selected if they satisfied all of the following criteria:

1) patients were pathologically diagnosed with cancer;
2) sufficient information was provided to estimate the relation between TRIM59 expression and the hazard ratios (HRs) of tumor patients;
3) when generating HRs from published Kaplan–Meier curves, the reported minimum and maximum follow-up times and number of subjects in each arm were reported.

### 2.3 Quality assessment

Two investigators (Chao and Wang) independently assessed the eligible studies in accordance with the Newcastle-Ottawa Quality Assessment Scale (NOS).^[8]^ The final score was the minimum grade.

### 2.4 Data extraction

Two authors (Chao and Wang) independently extracted the following data:

1) first author, publication year, country, sample size, methods, duration of follow-up, and other relevant data;
2) HRs and their 95% confidence intervals (CIs) for overall survival (OS) were extracted from the studies if the data were explicitly supplied in the published results – if the survival data were not provided, we extracted them from Kaplan–Meier curves for further processing;
3) the detection biomarker was TRIM59.

### 2.5 Statistical analysis

We used the HRs with their 95% CIs to estimate the effect of TRIM59 expression on survival rates. We directly extracted HRs with corresponding 95% CIs if they were provided in the original articles. Otherwise, the methods described by Parmar et al^[9]^ were used for HR estimation. The inconsistency index I^2^ was utilized to assess the heterogeneity of included studies. If *P* > .10 and I^2^ < 50%, we considered the heterogeneity non-significant, and a fixed-effect model was adopted; otherwise, a random-effects model was adopted. To estimate the reliability of the pooled results, a sensitivity analysis was performed by omitting single studies. In order to assess the publication bias, Begg and Egger funnel plots were drawn. Data management and analysis were performed with STATA 12.0 software (Stata Corporation, College Station, TX).

## 3 Results

### 3.1 Search results

As shown in the Flow Diagram, 229 studies were identified according to the search strategy from PubMed, Embase, VIP, CNKI and Wanfang Data. Of these, 73 studies were removed due to duplication. By screening the abstracts, 147 studies were excluded as they were laboratory research reports, contained irrelevant content, or were reviews. After reading through the full text, three additional studies were excluded due to a lack of sufficient data. Finally, 6 studies were included for further analysis.^[10–15]^

**Figure:**
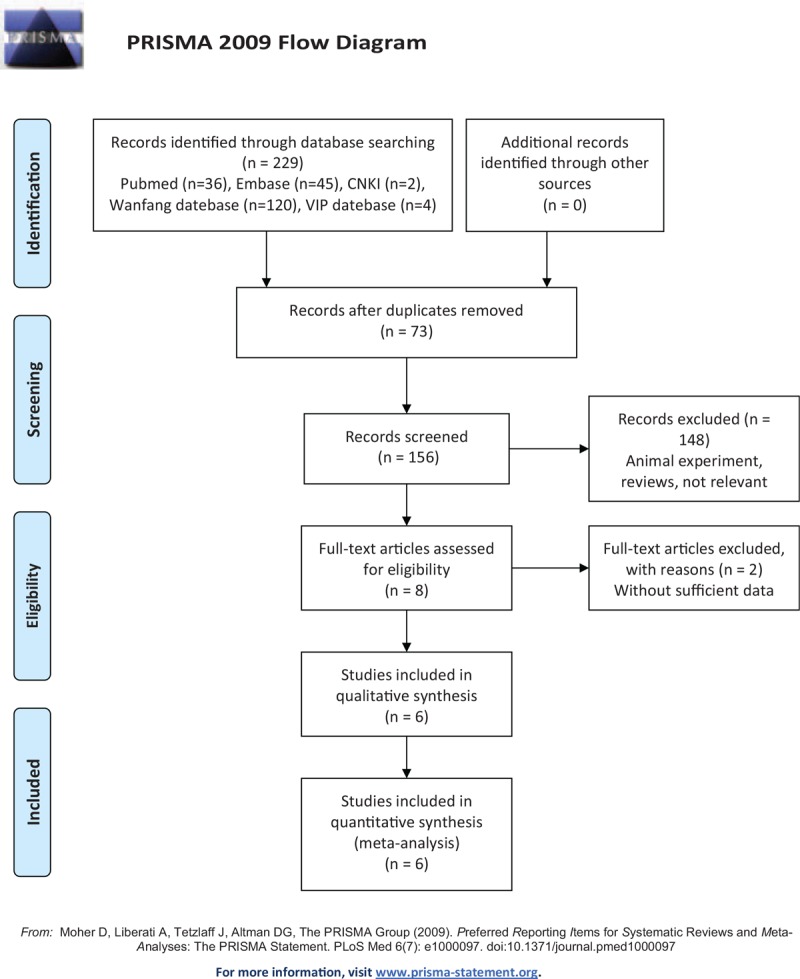


### 3.2 Study characteristics

The detailed information of each study is summarized in Table 1. The 6 studies were published between 2014 and 2018, and were all conducted in China. A total of 1584 patients were included, and the treatment strategy was surgery alone. The detection method used was immunohistochemistry (IHC). Five studies provided Kaplan–Meier curves,^[10,12–15]^ and one study reported the HR data directly.^[11]^ The quality scores ranged from 7 to 8.

**Table 1:**
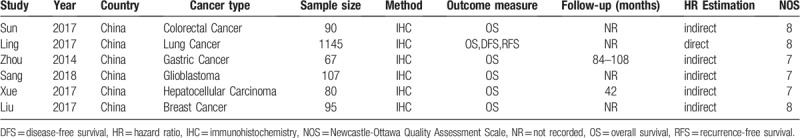
Basic characteristics of the included studies.

### 3.3 Correlation between TRIM59 expression and OS of cancer patients

A fixed- effect model was adopted due to the low level of heterogeneity among studies (I^2^ = 0, *P* = .744). A forest plot revealed that high expression levels of TRIM59 correlate with poor OS in tumor patients (HR = 1.43, 95%CI: 1.24–1.66, *P* < .001). Meanwhile, Heterogeneity analysis revealed that there was no between-study heterogeneity in the eight studies (Chi-squared = 2.71, d.f. = 5, *P* = .744, I^2^ = 0).

### 3.4 Sensitivity analysis

**Figure 1:**
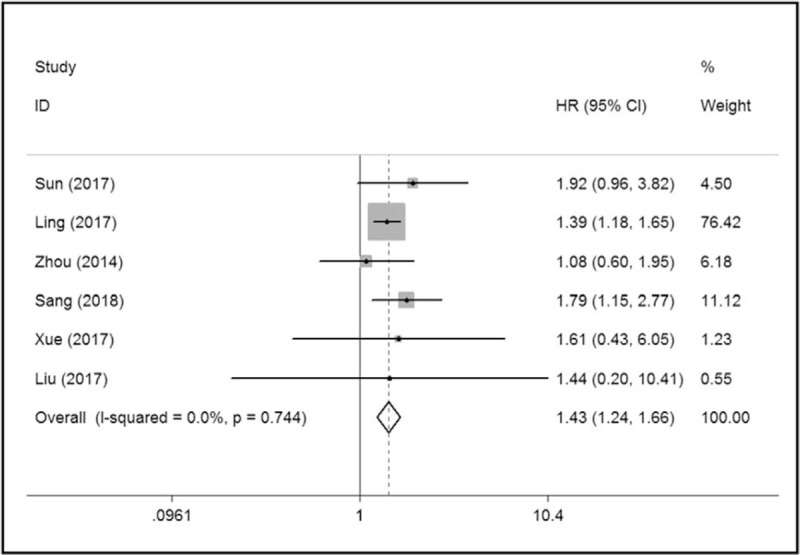
Forest plot of the association between TRIM59 and overall survival in solid tumors, HR = hazard ratio, CI = confidence interval.

**Figure 2:**
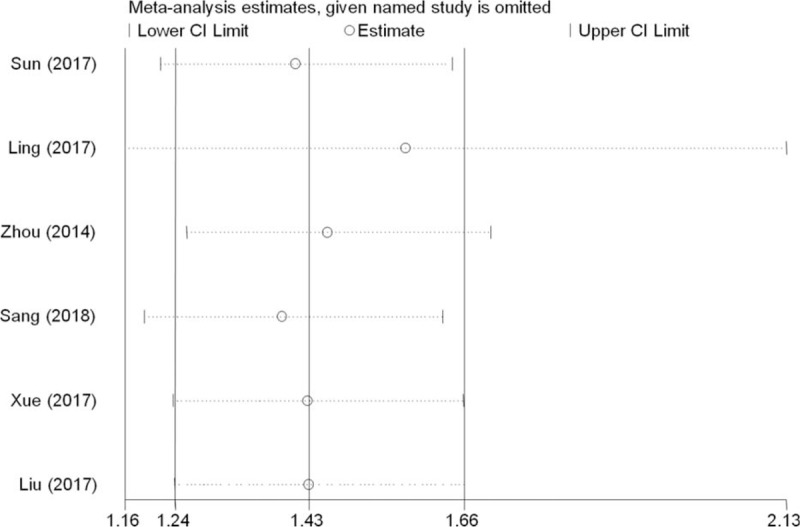
Sensitivity analysis of the relationships between TRIM59 and OS in solid tumor patients, CI = confidence interval, OS = overall survival.

Sensitivity analysis was performed to estimate the reliability of pooled results by omitting single studies. As shown in Figure 1, the results showed that the pooled HR did not significantly change upon exclusion of any individual studies, meaning that the results of this meta-analysis are stable (Fig. 2).

### 3.5 Publication bias

**Figure 3:**
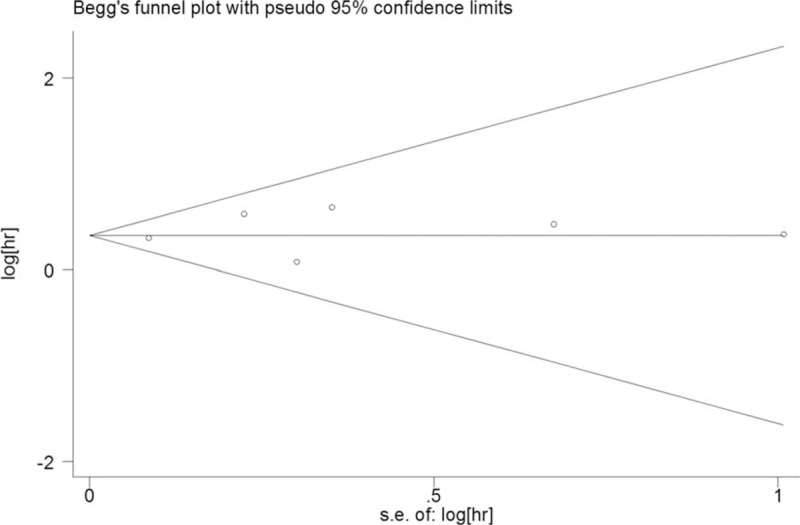
Begg funnel plot indicator test for publication bias of TRIM59 and overall survival, HR = hazard ratio SE = standard error.

We constructed a Begg funnel plot with pseudo 95% confidence limits and performed an Egger test to assess the publication bias of the included studies. Results showed that no publication bias was observed for OS (Begg test: *P* = 1 and Egger test: *P* = .491; Fig. 3). The figure indicated the consistency of the result.

## 4 Discussion

Tripartite motif (TRIM) family proteins have various functions in cellular processes, including intracellular signaling, development, apoptosis, protein quality control, innate immunity, autophagy, carcinogenesis, and angiogenesis. Research surrounding the TRIM59 protein has primarily focused on intracellular signaling, autophagy, and carcinogenesis. In dsDNA receptor-mediated signaling, TRIM59 binds to ECSIT (evolutionarily conserved signaling intermediate in Toll pathway), leading to the inhibition of NFkB (nuclear factor-kappa B) and IRF3 (Interferon regulatory factor 3) signaling.^[5]^ In terms of intracellular signaling and its role as an oncoprotein, TRIM59 may act by activating the PI3K/AKT (phosphatidylinositol-3-kinases/ protein-serine-threonine kinase) signaling pathway to induce migration and invasion in medulloblastoma.^[16]^ In support of this, silencing TRIM59 inhibits the TGF-β/Smad2/3 signaling pathway, leading to inhibited invasion and migration in bladder cancer cells.^[17]^ A series of studies have indicated that many TRIM proteins are linked to autophagy.^[18–21]^ TRIM59 regulates autophagy by modulating both the transcription and the ubiquitination of BECN1 (Beclin 1).^[22]^ An increasing number of studies have reported that TRIM59 is closely related to carcinogenesis and tumor prognosis. In 2011, TRIM59 was initially found to act as a potential oncoprotein in a mouse model of cancer. In basic laboratory research, TRIM59 has been shown to promote the migration and invasion of cancer cells, and knockdown of TRIM59 inhibits malignancy processes in human cancer cells. Moreover, TRIM59 has been shown to be involved in the cytotoxicity of BCG (Bacillus Calmette-Guerin)-activated macrophages.^[23]^ In human clinical studies, overexpression of TRIM59 has been found in colorectal cancer, breast cancer, hepatocellular carcinoma, lung cancer, gastric cancer, glioblastoma and others. Additionally, many studies have investigated the specific association of TRIM59 with tumorigenesis in each respective cancer field. We therefore conducted this meta-analysis to obtain a more definitive conclusion on the relationship between TRIM59 and cancer-related survival.

With the advances in the study of TRIM59, researchers have found that TRIM59 is involved in a variety of malignant biological behaviors in tumors, which suggests that TRIM59 has the potential to be a target for future cancer therapies. As far as we know, this is the first meta-analysis to investigate the prognostic role of TRIM59 in tumor patients. Our analysis revealed that high expression of TRIM59 is associated with poor prognoses in cancer patients. The six articles included in this study indicated that TRIM59 was related to TNM (Tumor Node Metastasis) in hepatocellular carcinoma, lung cancer, colorectal cancer, and breast cancer. Because of data limitations, we could not further analyze the association between TRIM59 and TNM. We look forward to future studies and original data concerning the relationship between TRIM59 and tumor biology.

Although the number of studies included was only six, the number of patients included was 1584, which does not influence the reliability of results. The pooled HR value was 1.43 (95%CI: 1.24–1.66, *P* < .001), which did not change when single studies were omitted, which demonstrates the consistency of the results. Additionally, we found no heterogeneity between studies.

## 5 Limitations

There are some limitations to this meta-analysis:

1) the number of included studies and the total sample size were limited. Only 6 types of tumor were included, thus we were unable to conduct a subgroup analysis.
2) The articles included were only from China, therefore results may only be applicable to Asian populations. An increasing number of studies, especially including European and American patient populations should be included.
3) There was insufficient data to investigate the association between clinico-pathological characteristics and the level of TRIM59.
4) A lack of unified cut-off criteria for TRIM59 levels made it difficult to distinguish between patients with high vs. low TRIM59 expression.
5) The HR was obtained using the Engauge Digitizer 10.0 software from the Kaplan–Meier curve, which may be a source of error.
6) The search model might have excluded potentially eligible studies that were written in other languages.

## 6 Conclusion

This meta-analysis revealed that high TRIM59 expression is a predictor of poor OS in cancer patients. TRIM59 may therefore serve as a promising prognostic biomarker that can be utilized to improve future cancer therapies.

## Author contributions

**Conceptualization:** Min Wang, Bin Wang.

**Data curation:** Min Wang, Ce Chao.

**Formal analysis:** Min Wang, Ce Chao.

**Funding acquisition:** Min Wang, Ce Chao, Guanghua Luo, Xianghong Zhan, Dongmei Di.

**Investigation:** Min Wang, Yongxiang Qian.

**Methodology:** Min Wang, Guanghua Luo, Bin Wang.

**Project administration:** Bin Wang, Xiaoying Zhang.

**Resources:** Min Wang.

**Software:** Ce Chao, Bin Wang.

**Supervision:** Bin Wang, Xianghong Zhan, Dongmei Di, Yongxiang Qian.

**Validation:** Xiaoying Zhang.

**Writing – original draft:** Ce Chao.

**Writing – review & editing:** Guanghua Luo, Bin Wang, Yongxiang Qian, Xiaoying Zhang.
